# Adjunctive Interferential Electrotherapy Provides Transient Postoperative Analgesia After Hemilaminectomy in Dogs

**DOI:** 10.3390/ani16101452

**Published:** 2026-05-09

**Authors:** Lizeth A. Montano Garibay, Gabriel A. Garcia, Erin Miscioscia, Jennifer A. Repac, James Colee, Christina Montalbano

**Affiliations:** 1 Department of Comparative, Diagnostic & Population Medicine, University of Florida College of Veterinary Medicine, Gainesville, FL 32608, USA; emiscioscia@ufl.edu (E.M.); jrepac@ufl.edu (J.A.R.); 2 Small Animal Clinical Sciences, University of Florida College of Veterinary Medicine, Gainesville, FL 32608, USA; gabrielagarcia@ufl.edu; 3 Consultant, Statistical Consulting Unit, University of Florida Institute of Food and Agricultural Sciences, Gainesville, FL 32608, USA; colee@ufl.edu

**Keywords:** interferential electrotherapy, postoperative pain, hemilaminectomy, intervertebral disc extrusion, canine rehabilitation, multimodal analgesia

## Abstract

In dogs undergoing hemilaminectomy for acute intervertebral disc extrusion (IVDE), adjunctive nonpharmacologic therapies may help provide postoperative analgesia while reducing reliance on opioids and their associated adverse effects. This prospective, randomized, blinded, controlled trial evaluated the short-term analgesic effects of interferential current electrotherapy (IFC) in dogs following thoracolumbar hemilaminectomy for acute IVDE. Twenty dogs were randomly assigned to receive conventional analgesia alone (control, *n* = 10) or conventional analgesia plus IFC (*n* = 10). The IFC group received three IFC sessions administered within the first 24 h postoperatively. Outcome measures were collected at three predefined postoperative time points corresponding to IFC treatment sessions: T1 (2–4 h), T2 (8–18 h), and T3 (20–24 h postoperatively). Outcome measures included Modified Glasgow Composite Pain Scale–Short Form (CMPS-SF) scores, total postoperative opioid consumption, and peri-incisional mechanical sensory thresholds. Dogs treated with IFC demonstrated significantly lower CMPS-SF pain scores at the T2 time point compared with controls (*p* = 0.03); however, no differences were observed at T1 or T3 time points. Total opioid consumption and mechanical sensory thresholds did not differ significantly between groups at any time point. These findings suggest that IFC may provide transient postoperative analgesic benefit but does not appear to reduce opioid requirements or mechanical sensory thresholds within the first 24 h following hemilaminectomy. Further studies with larger sample sizes are warranted to better define the role of IFC in multimodal postoperative pain management and to explore optimal treatment protocols.

## Simple Summary

Back pain caused by a ruptured spinal disc is a common condition in dogs and often requires surgery. Although surgery can help improve recovery, many dogs experience pain afterward that needs to be managed carefully. Pain medications such as opioids are commonly used, but they can cause side effects, so veterinarians are interested in finding additional ways to improve comfort. Interferential current therapy is a type of electrical stimulation used in rehabilitation that may help reduce pain. In this study, we explored whether adding this therapy to standard care could improve comfort in dogs after spinal surgery. Dogs that received this treatment appeared more comfortable at one point during recovery, suggesting it may provide short-term pain relief. While the overall impact was limited, this therapy may still be a helpful addition to pain management strategies. Further studies are needed to better understand how and when it is most effective.

## 1. Introduction

Intervertebral disc extrusion (IVDE) particularly affecting the thoracolumbar spinal region is one of the most common neurologic disorders encountered in small animal veterinary practice and represents a leading cause of acute spinal cord injury in dogs [1,2,3,4]. IVDE most commonly affects young to middle-aged chondrodystrophic breeds, although non-chondrodystrophic dogs may also be affected [2,3,4]. Clinical presentation spans a broad spectrum, ranging from spinal hyperesthesia to severe neurologic dysfunction, including paraplegia with loss of deep pain perception. Management options include conservative therapy consisting of strict activity restriction, analgesia, anti-inflammatory medications, and rehabilitation, or surgical decompression to alleviate spinal cord compression. In acute cases with significant neurologic compromise, surgical intervention, most commonly hemilaminectomy, is frequently recommended to remove extruded disc material, facilitate neurologic recovery, and optimize functional outcomes [1].

Despite ongoing advances in surgical techniques, effective pain management following IVDE surgery remains a significant clinical challenge [5,6,7]. Standard analgesic protocols typically employ a multimodal pharmacologic approach that includes non-steroidal anti-inflammatory drugs or corticosteroids, opioids, and adjunctive agents such as gabapentin. Although generally effective, reliance on opioid-based analgesia has been associated with adverse effects, increased hospitalization costs, and prolonged length of stay in small animal surgical patients [6,7,8]. Common opioid-related side effects in dogs include sedation, respiratory depression, dysphoria during recovery, nausea, and decreased appetite. These complications may hinder postoperative management and early rehabilitation. Consequently, opioids are often reserved for breakthrough or “rescue” analgesia as needed, underscoring the need for safe and effective adjunctive, non-pharmacologic analgesic modalities to optimize postoperative comfort and recovery [5,8].

Electrotherapy has been increasingly explored as a complementary approach for pain control. Transcutaneous electrical nerve stimulation (TENS) has demonstrated analgesic efficacy in both experimental and clinical settings, with studies in both people and dogs reporting reduced pain intensity compared with control or placebo interventions [9,10,11,12]. The analgesic effects of TENS depend on stimulation parameters, particularly the therapeutic pulse frequency [11,12]. High-frequency TENS (HF-TENS; typically >50 Hz) is thought to reduce pain primarily through activation of large-diameter Aβ mechanoreceptive afferents, consistent with the gate control theory of pain, thereby inhibiting nociceptive transmission at the spinal cord level [11,12]. In contrast, low-frequency TENS (LF-TENS; typically <10 Hz) is associated with activation of smaller diameter afferent fibers and stimulation of descending inhibitory pathways, promoting the release of endogenous opioids such as endorphins and enkephalins [11,12]. TENS may also influence inflammatory processes through modulation of proinflammatory cytokines [13]. Despite these mechanisms, considerable heterogeneity in stimulation parameters, treatment duration, and patient populations has been reported across studies, resulting in inconsistent findings regarding the relative efficacy of HF- and LF-TENS and limiting direct clinical translation [11,13,14].

Interferential current (IFC) is a form of electrotherapy that differs from conventional TENS in both frequency characteristics and mode of delivery [15,16,17]. IFC uses two independent medium-frequency alternating currents (typically in the kilohertz range, e.g., ~4000 Hz) delivered through separate electrode pairs [17,18]. These currents intersect within the tissues to generate an amplitude-modulated low-frequency “beat” effect corresponding to the difference between the two frequencies [18,19]. The use of medium-frequency currents reduces skin impedance, allowing deeper penetration of the electrical stimulus and potentially improving patient comfort compared with low-frequency stimulation [15,16,17].

The distribution and characteristics of the electrical stimulus produced by IFC are influenced by electrode placement, tissue impedance, and nerve fiber orientation [17]. At the point of intersection, the interacting currents may reinforce each other, producing a modulated waveform; however, the degree of modulation varies depending on nerve fiber orientation relative to the electrical field, with some fibers receiving fully modulated stimulation and others experiencing partially modulated or unmodulated currents [15]. As a result, the actual stimulus delivered to neural structures can vary depending on anatomical and technical factors, which may influence treatment consistency and clinical outcomes. In human medicine, IFC has been proposed to provide superior analgesic effects compared with traditional TENS, particularly for deeper tissues, although findings remain variable across studies [16,17,19,20].

Although electrotherapy modalities are widely used in veterinary rehabilitation, there remains a paucity of controlled clinical studies evaluating their efficacy and defining evidence-based protocols in dogs and cats [21]. Previous veterinary studies investigating IFC have largely focused on chronic conditions such as osteoarthritis, ankylosing spondylitis, and paraparesis, with reported improvements in chronic pain or functional outcomes [22,23,24]. Whether IFC provides meaningful benefit for acute postoperative pain remains unknown, and no studies have specifically evaluated its use following hemilaminectomy for IVDE [21,22,23,24].

Accurate assessment of postoperative pain in dogs requires the use of both validated subjective and objective outcome measures, as pain cannot be directly self-reported. Subjective pain scales are commonly used for the evaluation of acute and postoperative pain because they systematically assess behavioral and physiologic indicators of discomfort. The Glasgow Composite Measure Pain Scale–Short Form (CMPS-SF) is a previously validated and widely utilized instrument for acute pain assessment in dogs and is frequently employed in postoperative analgesia studies to guide clinical decision-making [25,26]. In addition to subjective scoring, mechanical sensory threshold (MST) testing using pressure algometry provides an objective, quantitative assessment of nociceptive sensitivity and has demonstrated reliability in human pain research [27], and has been shown to capture changes in mechanical sensitivity in dogs with acute thoracolumbar intervertebral disc extrusion, including in the perioperative period following surgical decompression [28,29]. The combined use of subjective pain scales and objective MST assessment allows for a more comprehensive evaluation of postoperative pain and treatment response.

Given the high prevalence of IVDE, increasing owner interest in complementary postoperative therapies, and the relative accessibility of electrotherapy devices, rigorous evaluation of IFC in this setting is warranted. However, despite its use in veterinary rehabilitation and investigation in chronic conditions, there is a lack of evidence evaluating its efficacy for acute postoperative pain, particularly following hemilaminectomy in dogs with thoracolumbar IVDE.

The objective of this study was to evaluate the effect of adjunctive interferential current therapy on postoperative pain in dogs undergoing hemilaminectomy. We hypothesized that interferential current therapy would provide improved short-term analgesia compared with conventional analgesia alone.

## 2. Materials and Methods

### 2.1. Study Design

This study was approved by the University of Florida Institutional Care and Use Committee (IACUC Approval # IACUC202400000084 approved 18 March 2024) and by the College of Veterinary Medicine Hospital Research Review Committee (VHRCC Approval # 2024-30 approved 16 September 2024). This study was designed as a prospective, randomized, controlled clinical trial conducted at the University of Florida Veterinary Hospital from November 2024 to July 2025.

A priori sample size estimation indicated that 10 dogs per group would be required to achieve a statistical power of 0.80 with a Type I error rate (α) of 0.05 to detect a difference in MST between groups. MST was selected for sample size estimation because it has less variability compared to subjective pain scores.

### 2.2. Inclusion Criteria

Client-owned dogs undergoing hemilaminectomy for acute thoracolumbar IVDE (T3–L3) were eligible for enrollment. Dogs were included if they were between 2 and 10 years of age and weighed between 4 and 30 kg, reflecting the typical signalment of dogs undergoing surgical treatment for thoracolumbar IVDE in clinical practice and minimizing variability associated with extreme age or body size. Eligible dogs were required to have intact deep pain perception at the time of enrollment. Diagnosis of acute thoracolumbar IVDE was established by magnetic resonance imaging (MRI) or computed tomography (CT) and confirmed intraoperatively by a board-certified neurologist or neurology resident. Only dogs that underwent a standard T3–L3 hemilaminectomy without intraoperative or immediate postoperative complications were included.

Dogs were excluded if they were excessively fearful, nervous, or aggressive such that physical examination or IFC treatment could not be safely performed. Additional exclusion criteria included concurrent spinal cord or neurologic disease, dermatologic conditions interfering with electrode placement, or absence of deep pain perception.

### 2.3. Group Allocation and Blinding

Following surgery, dogs were randomly assigned to either the IFC treatment group or the control group until 10 dogs completed the study in each group. Group allocation was performed using a pre-generated randomized (random.org) list of 30 numbers to allow replacement in the event of study withdrawal or postoperative complications. Personnel responsible for pain scoring, opioid administration, and outcome assessments were blinded to treatment allocation. Dogs in the control group were removed to a separate room for the same duration and at the same postoperative time points as dogs receiving IFC to maintain blinding.

### 2.4. Analgesic Management

All dogs received an individualized multimodal analgesic protocol. The preoperative analgesic plan, including administration of a retromammillary local anesthetic block, was determined by a board-certified anesthesiologist or anesthesia resident. The retromammillary block was performed preoperatively using bupivacaine 0.5% (2.5 mg/kg) combined with dexmedetomidine (0.5 µg/kg), diluted to a final concentration of 1 µg/mL. Postoperative analgesic management was determined by the neurology resident surgeon based on clinical assessment and pain scores.

Opioid administration was guided by pain severity using the Short Form of the Modified Glasgow Composite Pain Scale (CMPS-SF). Rescue analgesic intervention was triggered by a CMPS-SF score > 5 [26]. Methadone was the primary rescue opioid administered, given as intermittent intravenous boluses, with dose and frequency tailored to individual patient needs based on pain scores. Use of a fentanyl continuous rate infusion (CRI) was permitted if clinically indicated, and all opioid administration, including methadone and fentanyl, was recorded. Additional medications, including gabapentin and non-steroidal anti-inflammatory drugs or corticosteroids, were permitted at clinician discretion and documented.

Cold compresses were permitted as part of routine postoperative care except during the two-hour periods before and after IFC treatments.

### 2.5. Interferential Current Therapy

Dogs assigned to the treatment group received IFC therapy in addition to conventional analgesia. IFC treatments were initiated once dogs were alert following anesthesia and able to undergo baseline pain scoring and MST testing.

Three IFC sessions were administered during the first 24 h following surgery and were defined as sequential postoperative treatment time points (T1–T3), with a minimum interval of 6–8 h between treatments.

The first IFC session (T1) was delivered within 2–4 h postoperatively, the second session (T2) within 8–18 h, and the third session (T3) within 20–24 h postoperatively. Exact treatment timing varied based on patient recovery, hospital workflow, and clinical considerations. Treatment windows were intentionally flexible to reflect real-world clinical practice and to enhance the feasibility and reproducibility of the protocol.

**Scheme 1:**
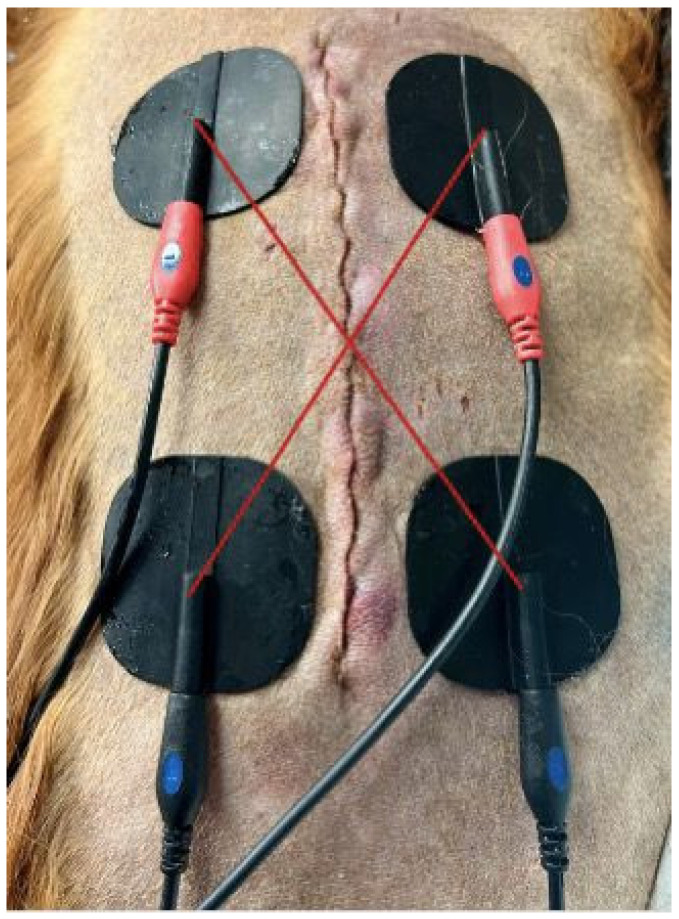
Electrode placement on a patient.

IFC therapy was delivered using an electrical stimulator (Intelect^®^ Vet, Chattanooga, TN, USA) per manufacturer’s recommendations and as demonstrated in human studies [18]. Four silica carbon reusable square electrodes (Amrex^®^, Paramount, CA, USA) were used, with electrode size 1.7″ × 1.5″ (4.3 cm × 3.8 cm). Electrodes were placed diagonally in an “X” configuration, cranial and caudal to the surgical site, with spacing of no more than one to two vertebral bodies and a maximum cranial–caudal distance of 20 cm (Scheme 1). Lubricating sterile gel (Medichoice^®^, Medline Industries, Inc., Northfield, IL, USA) was used to conduct the current in convenient single-use foil packets (3 g). A total of 4 packets were used to apply 3 g to each electrode.

IFC parameters were as follows: an interferential waveform with constant voltage, a carrier frequency of 4000 Hz, a beat frequency of 80/150 Hz, an automatic vector scan set at 40%, and a treatment duration of 20 min. Treatment intensity was gradually increased from baseline until visible muscle fasciculation or a patient response was observed, then adjusted to the highest level tolerated without signs of distress, including head turning toward the treatment site, biting or snapping, increased anxiety or agitation, panting, vocalization (e.g., whining), or attempts to move away. All IFC treatments were administered by a single investigator (LM) to ensure consistency.

### 2.6. Sham Control Treatment

Dogs in the control group underwent a sham treatment with placement of the sterile conduction gel and electrodes connected to the electrical stimulator machine with the machine turned off for the same 20 min duration as the IFC group. All sham treatments were administered by the same single investigator (LM).

### 2.7. Outcome Measures

#### 2.7.1. Pain Assessment

Pain was assessed using the CMPS-SF. Because mobility could not be meaningfully assessed in dogs following spinal surgery, the mobility section was omitted in accordance with published CMPS-SF guidance, resulting in a maximum possible score of 20 and an analgesic intervention threshold of >5 [26]. The CMPS-SF is routinely used in our hospital for postoperative pain assessment, and all intensive care unit (ICU) and progressive care ward (PCW) technicians are trained and familiar with its application. The initial pain score was obtained once the dog was awake, conscious, and no longer affected by anesthetic dissociation and was performed by a blinded ICU or PCW veterinary technician. Subsequent pain assessments were performed every four to six hours by ICU or PCW technicians who were blinded to group assignments. Pain assessments were performed according to routine postoperative intervals independent of IFC administration. For statistical analysis, CMPS-SF values corresponding to T1, T2, and T3 were defined as the assessment occurring closest in time to each predefined IFC postoperative window (or the corresponding matched postoperative window in control dogs).

#### 2.7.2. Opioid Consumption

Total postoperative opioid use was recorded using a standardized tracking table and verified through the electronic medical record. Postoperative opioid consumption was quantified based on rescue methadone administration within the first 24 h following surgery and normalized to body weight (mg/kg) to allow comparison between groups.

#### 2.7.3. Mechanical Sensory Threshold Testing

**Scheme 2:**
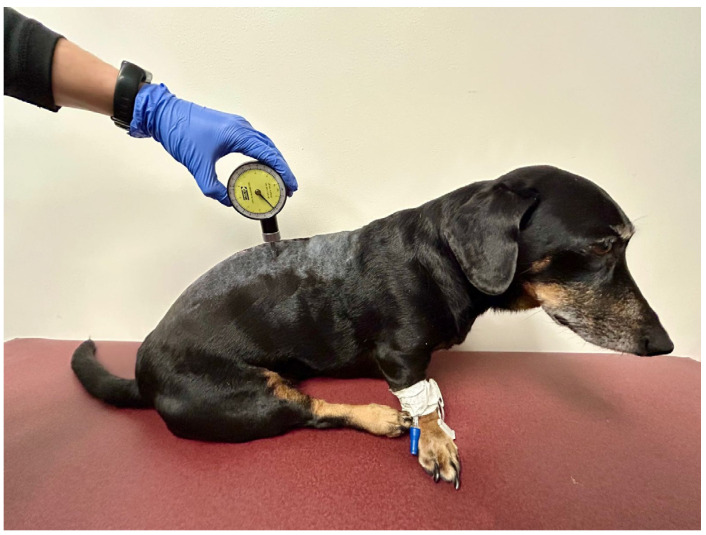
MST measurement in a patient. Representative example demonstrating pressure algometry performed at the peri-incisional test site. The measurement site is located on the right side at the thoracolumbar junction, approximately 3–5 mm lateral to the dorsal midline, adjacent to but not directly over the surgical incision.

MST was measured using a commercially available pressure algometer (Wagner FDK/FDN Force Dial^®,^ Wagner Instruments, Greenwich, CT, USA), a handheld force-testing device with a 6 mm flat compression head. Measurements were obtained at two standardized locations: a peri-incisional site near the thoracolumbar junction and a distant control site at the T1–T3 region (Scheme 2). The peri-incisional site was located on the same side as the surgical lesion, positioned 3–5 mm lateral to the dorsal midline, adjacent to but not directly over the surgical incision. The control site was similarly positioned on the same side of the lesion and 3–5 mm lateral to the dorsal midline at the T1–T3 level to maintain consistency in laterality and proximity to midline while avoiding the surgical field. MST testing was conducted immediately prior to each treatment session and again within 30–60 min following treatment by the same blinded observer (CM). All measurements were performed with dogs positioned in a standardized sitting posture, as body position has been shown to influence mechanical threshold responses and repeatability in dogs [29]. The observer sat quietly behind the dog and out of the patient’s field of view, providing minimal restraint to reduce anticipatory or visual cue-related responses. Pressure was applied at a consistent rate until a behavioral response was observed (avoidance, head turning, vocalization, or attempt to bite) or until a predetermined maximum force of 11 pounds was reached.

Each measurement was performed in triplicate with one-minute intervals between trials, and the mean value was calculated for analysis. To reduce observer bias, roles were strictly separated. The blinded investigator (CM) applied the pressure stimulus and was responsible solely for detecting the predefined behavioral response indicating nociceptive threshold. The pressure algometer was positioned such that CM could not view the force display at any time. Once the behavioral response occurred, the unblinded investigator (LM) recorded the corresponding force value (in lbs). The blinded investigator did not view, dictate, or record any numeric measurements. Triplicate measures were averaged at each timepoint prior to statistical analysis.

#### 2.7.4. Statistical Analysis

Demographic variables were summarized descriptively and compared between groups. Continuous variables (age and body weight) were reported as median (range) and compared using the Mann–Whitney U test, while categorical variables (sex) were compared using Fisher’s exact test.

Gabapentin doses (mg/kg) were summarized as median (range) and compared between groups using the Mann–Whitney U test. Anti-inflammatory medication class (NSAID vs. corticosteroid) was compared between groups using Fisher’s exact test.

CMPS-SF scores were analyzed using a linear mixed-effects model. Although CMPS-SF is an ordinal scale, scores were analyzed using a linear mixed-effects model and are presented as model-adjusted means ± standard error to allow evaluation of treatment effects over time. This approach facilitated estimation of group differences across repeated measures and aligns with previous veterinary studies utilizing this instrument. Time point (T1, T2, and T3), treatment group (IFC vs. control), and their interaction were included as fixed effects, with dog included as a random effect to account for repeated measures. Model adjusted least squares means were estimated and compared between treatment groups at each time point.

MST were analyzed as the difference in pressure calculated as post-treatment minus pre-treatment values at both the peri-incisional test site and the distant control site using a linear mixed-effects model. Test site (peri-incisional site vs. distant control site), treatment group, and their interaction were specified as fixed effects, with dog included as a random effect. Variability in MST measurements across sites and treatment phases was evaluated to assess the potential influence of repeated testing or behavioral anticipation.

Total postoperative opioid consumption within the first 24 h following surgery was evaluated based on rescue methadone administration normalized to body weight (mg/kg). Postoperative opioid consumption was compared between treatment groups using the Wilcoxon two-sample nonparametric test. A nonparametric approach was selected due to the skewed distribution of opioid use, with a substantial proportion of dogs not requiring rescue opioid administration.

Statistical significance was set at *p* < 0.05 for all analyses.

## 3. Results

### 3.1. Study Population

Twenty-one dogs were initially enrolled in this study. One dog was excluded after enrollment due to the absence of deep pain perception on post-operative neurologic examination, leaving a total of 20 dogs that completed the study. All dogs completed the planned postoperative assessments during the treatment period.

Of the 20 dogs included in the final analysis, breeds included Welsh Corgi, Dachshund, Skye Terrier, French Bulldog, Russell Terrier, Labrador mix and mixed breed dogs. 10 dogs were assigned to the IFC treatment group, and 10 dogs were assigned to the control group. Dogs in the IFC group had a median age of 6.5 years (range 2–10 years) and a median body weight of 8.15 kg (range 4.0–12.2 kg); 7 were spayed female and 3 were neutered male. Dogs in the control group had a median age of 7.0 years (range 2–10 years) and a median body weight of 11.4 kg (range 4.93–30.0 kg), 5 were spayed female, 1 intact female, and 4 were neutered male. There was no significant difference in age or sex between groups. There was a significant difference in weight between groups (*p* = 0.03), with dogs in the control group being heavier than the treatment group.

Oral analgesics were administered at clinician discretion. All 20 dogs received gabapentin every 8 h; dogs in the IFC group received a median dose of 10.58 mg/kg (range 6.25–19.23), while dogs in the control group received a median dose of 10.15 mg/kg (range 7.30–16.26). Gabapentin dosing did not differ significantly between IFC-treated and control dogs (*p* = 0.73).

In the IFC group, 8 of 10 dogs received nonsteroidal anti-inflammatory drugs (NSAIDs; 6 received carprofen and 2 received meloxicam), while 2 dogs received neither an NSAID nor a corticosteroid. In the control group, 6 of 10 dogs received NSAIDs (all carprofen), 3 dogs received corticosteroids, and 1 dog received neither an NSAID nor a corticosteroid. Use of corticosteroids did not differ significantly between IFC-treated and control dogs (*p* = 0.21).

### 3.2. CMPS-SF Scores

**Figure 1:**
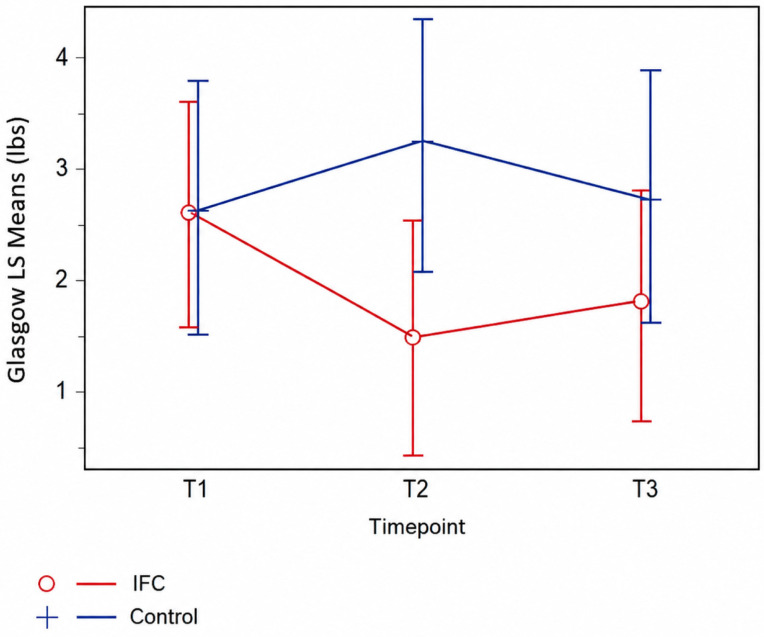
Model-adjusted Glasgow Composite Pain Scale–Short Form (CMPS-SF) scores over time in IFC-treated and sham-treated dogs. Values represent least squares means ± standard error derived from a linear mixed-effects model. IFC-treated dogs had significantly lower CMPS-SF scores at T2 compared with sham-treated dogs (*p* = 0.03). No significant between-group differences were detected at T1 or T3.

CMPS-SF scores are summarized in Table 1. Pain scores were comparable between groups at T1, indicating similar baseline postoperative pain levels (*p* = 0.97). At T2, dogs receiving IFC had significantly lower pain scores compared with the control group (*p* = 0.03). At T3, no statistically significant difference was detected between groups (*p* = 0.23). However, pain scores remained numerically lower in the IFC-treated dogs compared to controls (1.8 ± 0.51 vs. 2.75 ± 0.57). (Figure 1).

Within the control group, pain scores increased significantly from T1 to T2 (*p* = 0.02), whereas there was no significant difference between any time point within the IFC group.

### 3.3. Mechanical Sensory Thresholds

Model-adjusted mechanical sensory threshold values are presented in Table 2. No statistically significant differences in MST changes were detected between IFC-treated and control dogs at either the peri-incisional or distant control sites.

MST values decreased slightly at each time point immediately following IFC treatment and sham procedure in both groups, consistent with expected postoperative sensitivity; however, these changes were similar between groups. Greater variability in MST responses was observed in the IFC group, particularly at the distant control site, but this did not translate into statistically significant differences.

### 3.4. Postoperative Opioid Use

**Figure 2:**
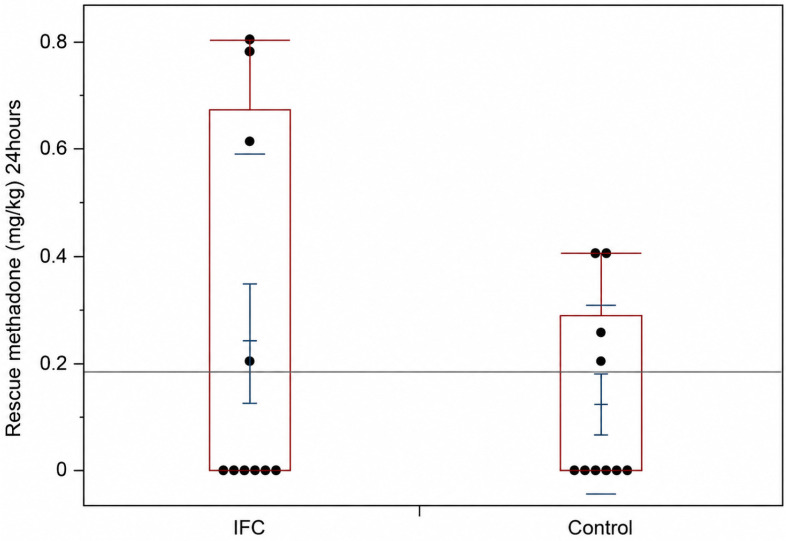
Postoperative rescue methadone consumption within the first 24 h following surgery in dogs receiving interferential current electrotherapy (IFC) or sham treatment. Values represent total rescue methadone dose normalized to body weight (mg/kg) for individual dogs. Black dots represent individual dogs. The central blue line represents the mean value ± 1 standard error (SE), and the outer blue lines represent ± 2 SE. The red box contains the middle 50% of the data (interquartile range), and the outer red lines represent the overall data spread (approximately 90–95% of observations). Rescue methadone use at 24 h was similar between IFC-treated and control dogs, with no statistically significant difference observed (Wilcoxon two-sample test, *p* = 0.76).

Postoperative opioid use was low and comparable between groups (Table 3, Figure 2). Methadone was the only opioid administered during the study period, and no dogs received additional opioid agents or continuous rate infusions.

Four of ten dogs in each group required rescue methadone, with no significant difference in total weight-normalized opioid consumption between groups (*p* = 0.76). Instances exceeding the predefined intervention threshold (CMPS-SF > 5) were infrequent, occurring in two dogs in the IFC group and one dog in the control group.

These findings indicate that IFC treatment did not reduce postoperative opioid requirements within the first 24 h following surgery.

## 4. Discussion

The results of this study suggest that IFC therapy, when used as an adjunct to conventional analgesia, may provide a transient analgesic benefit in dogs undergoing hemilaminectomy for acute thoracolumbar intervertebral disc extrusion. Dogs receiving IFC demonstrated lower postoperative pain scores at the T2 postoperative time point, while there was no effect at T1 or T3 time points. In contrast, no significant differences were detected in MST or postoperative opioid consumption between treatment groups. Collectively, these findings indicate that IFC may reduce subjective pain scores at a single postoperative time point but does not appear to significantly alter objective nociceptive sensitivity or opioid requirements within the first 24 h following surgery.

The absence of a detectable difference in pain scores between treatment groups at the earliest postoperative assessment (T1) was not unexpected and is likely attributable to the comprehensive perioperative analgesic protocols employed in this study. In the context of veterinary postoperative pain assessment, the immediate postoperative phase generally refers to the early recovery period within the first several hours following anesthetic recovery, during which residual effects of peri-operative analgesia are most pronounced. All dogs received multimodal analgesia, including locoregional anesthetic technique and peri-operative systemic opioids as part of standard surgical care. Residual effects of peri-operative analgesia likely provided substantial early postoperative pain control and thereby masked the incremental effects of IFC during this early recovery window. Similar observations have been reported in veterinary postoperative pain studies and within enhanced recovery after surgery (ERAS) frameworks, where adjunctive analgesic interventions do not demonstrate measurable benefits during the immediate postoperative phase but may show effects as perioperative analgesia wanes [5,25,26].

At the later postoperative T2 time treatment window, when the influence of perioperative analgesia is expected to decline, IFC-treated dogs demonstrated lower subjective pain scores compared with control dogs, consistent with a short-term analgesic effect. This temporal pattern aligns with findings from human clinical studies, in which IFC demonstrates analgesic effects at later post-intervention assessments, rather than immediately after procedure, when baseline analgesic effects are most pronounced [15,16,19,20]. While CMPS-SF scores at T3 were lower numerically in the treatment group, this finding was not statistically significant and should not be overinterpreted. However, because CMPS-SF was a secondary outcome and the study was powered for MST, the study may not have been adequately powered to detect differences in subjective pain scores across all postoperative time points. Meta-analyses evaluating IFC in musculoskeletal pain populations similarly describe time-dependent analgesic effects that are most evident in proximity to treatment sessions, rather than sustained pain reduction following treatment cessation [16,19,20]. Together, these observations support the interpretation that IFC may provide transient neuromodulation of ongoing nociceptive processing once baseline pharmacologic pain control diminishes, rather than acting as a source of sustained postoperative analgesia.

MST was included to provide an objective assessment of nociceptive sensitivity and to complement subjective pain scoring which reflects pain-related behaviors and patient responses. MST values demonstrated expected temporal changes following surgery, with lower thresholds observed at the peri-incisional site compared with the distant control site and modest changes over time. However, no statistically significant differences in MST changes were detected between treatment groups. These findings align with previous studies demonstrating that objective sensory testing and subjective pain scales assess related but distinct aspects of the pain experience [28,30]. In dogs undergoing hemilaminectomy for thoracolumbar intervertebral disc extrusion, correlations between CMPS-SF scores and mechanical thresholds have been reported to be weak to moderate, highlighting that localized sensitization and global pain-related behaviors may not change in parallel [28].

The lack of an IFC effect on MST in the present study may reflect the influence of perioperative analgesia, individual variability in sensory processing, and the relatively short observation period. In addition, mechanical threshold testing is known to be influenced by experimental protocol, testing conditions, and subject responsiveness, which can affect repeatability and sensitivity to change [27,29]. It is also possible that IFC primarily affects subjective pain perception rather than altering mechanical nociceptive thresholds, underscoring the importance of incorporating both subjective and objective outcome measures in postoperative pain research.

Postoperative opioid consumption during the first 24 h did not differ significantly between IFC-treated and control dogs. Rescue methadone use was infrequent in both groups, reflecting the relatively low overall requirement for supplemental opioid analgesia in this cohort. Similar findings have been reported in both veterinary and human studies evaluating adjunctive analgesic modalities, where improvements in pain scores were not consistently associated with reduced opioid use [6,7,8]. These results highlight the multifactorial nature of postoperative analgesic decision-making, which is influenced by patient behavior, anxiety, clinician interpretation, and institutional protocols, in addition to measured pain intensity [6,7].

The findings of the present study are consistent with prior veterinary investigations evaluating electrotherapy modalities for pain management. Studies assessing TENS in dogs with thoracolumbar hyperesthesia and musculoskeletal pain have reported reductions in pain scores during the treatment period and in the hours to days following intervention. However, sustained effects beyond the immediate or short-term postoperative period and consistent reductions in analgesic requirements have not been uniformly demonstrated [9,10]. Systematic reviews of veterinary electrotherapy further emphasize substantial heterogeneity in treatment protocols, outcome measures, and clinical indications, which limits direct comparison across studies and complicates interpretation of efficacy [21].

Most previous veterinary studies evaluating IFC have focused on chronic musculoskeletal conditions, such as osteoarthritis, in which pain mechanisms, central sensitization, and functional recovery trajectories differ markedly from those associated with acute postoperative neurologic injury [22,23,24]. For example, a 10-day treatment protocol with IFC has been shown to reduce pain and muscle atrophy in dogs with ankylosing spondylitis [22]. A single IFC treatment was shown to influence functional outcomes, including ground reaction forces, in dogs with chronic hip osteoarthritis [24]. The present study extends the existing literature by evaluating IFC in an acute postoperative spinal surgery population, thereby addressing an important gap and providing novel insight into its potential role in postoperative pain management following hemilaminectomy.

From a clinical perspective, the short-term reduction in pain scores observed in this study following IFC treatment may be relevant during early postoperative recovery. The flexible IFC protocol used in this study was intentionally designed to reflect real-world clinical practice, supporting feasibility, and external validity. Although IFC did not reduce opioid requirements, it may serve as a useful adjunct within a multimodal analgesic strategy aimed at improving patient comfort.

This study has several limitations. Patient heterogeneity may have influenced treatment responses. Although inclusion criteria were standardized, dogs in the control group had a higher mean body weight than those in the IFC group, largely due to a single dog at the upper end of the enrollment weight range. In addition, body condition score was not controlled, and differences in subcutaneous adipose tissue may have influenced current distribution and tissue penetration, potentially affecting individual responses to IFC. The relatively small sample size further limited statistical power to detect subtle differences between treatment groups, particularly for MST testing, and increased the possibility that baseline variability influenced the observed results (type II statistical error).

Although perioperative analgesic management was reflective of standard clinical practice, it was not fully standardized across dogs and may have introduced confounding variables. Multimodal analgesia included local anesthetic blocks, oral analgesics, rescue opioids, and anti-inflammatory medications, but the distribution of anti-inflammatory treatment was not equivalent between groups. In particular, corticosteroids were administered only in the control group, and not all dogs received anti-inflammatory therapy. Because NSAIDs and corticosteroids differ in mechanism of action and clinical effects, this imbalance reduces comparability between groups and makes it more difficult to isolate the specific effect of IFC on postoperative pain outcomes. Likewise, rescue opioid administration was guided by pain scoring but remained clinician dependent, which may have introduced additional variability. Collectively, these factors may have attenuated the ability to detect incremental effects of IFC or may have contributed to the differences observed at T2.

Variability in the timing of IFC administration may have further contributed to heterogeneity in individual treatment responses. In addition, postoperative pain assessment remains inherently complex; although a validated behavioral pain scale was used, pain-related behaviors may be influenced by anxiety, temperament, and environmental factors. Finally, surgical variables were not standardized and may also have affected postoperative pain and response to IFC. These included duration of clinical signs prior to surgery, operative time, number of decompression sites, and differences among surgeons performing the procedures. Therefore, the observed reduction in pain scores at T2 should be interpreted cautiously.

Future studies with larger sample sizes, more standardized perioperative analgesic protocols, and standardized IFC dosing parameters are warranted to better define the role of IFC in postoperative pain management. Extended follow-up periods, incorporation of additional objective outcome measures, and evaluation of patient demographics may help identify patient populations most likely to benefit from IFC as part of a multimodal analgesic approach. Given the limited availability of veterinary-specific data, much of the current understanding of interferential current therapy is extrapolated from human studies, which should be considered when interpreting these findings.

## 5. Conclusions

In conclusion, interferential current therapy was associated with a transient reduction in postoperative pain scores at a single time point in dogs undergoing hemilaminectomy for acute thoracolumbar intervertebral disc extrusion. This effect was not accompanied by changes in mechanical sensory thresholds or opioid consumption. These findings suggest that IFC may provide a modest, short-term adjunctive benefit as part of a multimodal analgesic strategy; however, its independent clinical impact remains uncertain. Further controlled studies with standardized protocols are required to better define its role in postoperative pain management.

## Figures and Tables

**Table 1:** Model-adjusted CMPS-SF pain scores (least squares mean ± SE) at T1, T2, and T3 in treatment and control groups.

Time	Treatment Group	Control Group	*p*-Value
T1	2.6 (0.51)	2.63 (0.57)	0.97
T2	1.5 (0.51)	3.25 (0.57)	0.03
T3	1.8 (0.51)	2.75 (0.57)	0.23

**Table 2:** Model-adjusted mechanical sensory threshold values (least squares mean +/−SE in lbs) at distant control and peri-incisional sites before and after treatment or sham control procedures.

	Treatment Group	Control Group
Control Site Pre	9.60 (0.53)	8.90 (0.55)
Control Site Post	8.51 (0.53)	8.73 (0.55)
Peri-incisional Site Pre	3.76 (0.53)	3.18 (0.55)
Peri-incisional Site Post	3.25 (0.53)	2.84 (0.55)

**Table 3:** Weight-normalized postoperative rescue methadone consumption within 24 h following surgery.

Group	Dose Receiving Methadone (*n*/*N*)	Total Dose (mg/kg), Median (Range)
IFC Treatment	4/10	0 (0–0.8)
Sham	4/10	0 (0–0.4)

## Data Availability

The data presented in this study are available from the corresponding author upon reasonable request.

## Abbreviations

The following abbreviations are used in this manuscript:

IVDE	Intervertebral disc extrusion
IFC	Interferential current electrotherapy
CMPS-SF	Modified Glasgow Composite Pain Scale-Short Form
TENS	Transcutaneous electrical nerve stimulation
MST	Mechanical sensory threshold
MRI	Magnetic resonance imaging
CT	Computed tomography
NSAID	Non-steroidal anti-inflammatory drug
ERAS	Enhanced recovery after surgery
